# Carrier Multiplication and Photoexcited Many-Body States in Solution-Processed 2H-MoSe_2_

**DOI:** 10.1021/acsnano.4c18254

**Published:** 2025-03-06

**Authors:** Goutam Ghosh, Tian Carey, Stevie Furxhiu, Sven Weerdenburg, Nisha Singh, Marco van der Laan, Susan E. Branchett, Sophie Jaspers, John W. Suijkerbuijk, Fedor Lipilin, Zdeněk Sofer, Jonathan N. Coleman, Peter Schall, Laurens D. A. Siebbeles

**Affiliations:** † Chemical Engineering Department, Delft University of Technology, Van der Maasweg 9, Delft 2629 HZ, The Netherlands; ‡ School of Physics, CRANN & AMBER Research Centres, Trinity College Dublin, Dublin D02 E8C0, Ireland; § Institute of Physics, University of Amsterdam, Science Park 904, Amsterdam 1098 XH, The Netherlands; ∥ ICT Innovation, Delft University of Technology, Landbergstraat 15, Delft 2628 CE, The Netherlands; ⊥ Department of Inorganic Chemistry, University of Chemistry and Technology Prague, Technická 5, Prague 6 166 28, Czech Republic

**Keywords:** carrier multiplication, excitons, trions, solution-processed transition metal dichalcogenide, transient absorption spectroscopy, terahertz spectroscopy

## Abstract

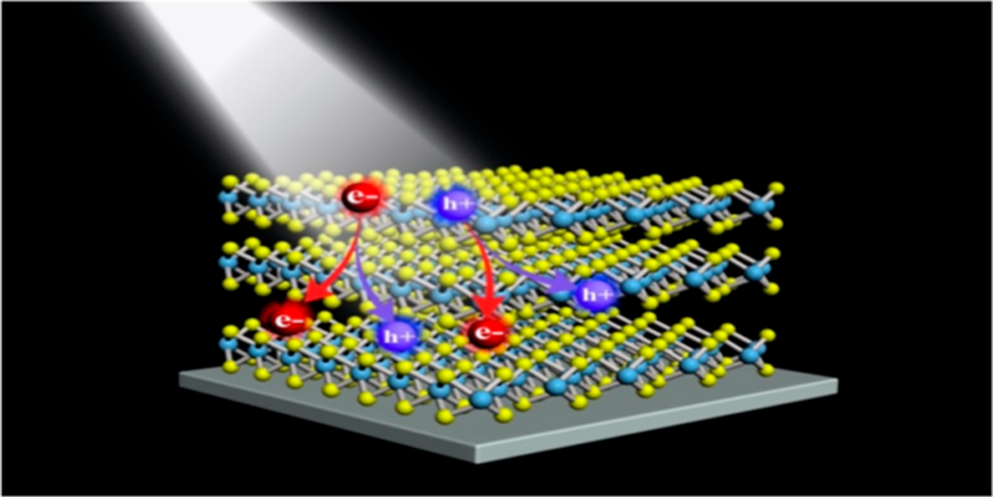

Carrier multiplication (CM), where a single high-energy photon generates multiple electron–hole pairs, offers a promising route to enhance the efficiency of solar cells and photodetectors.Transition metal dichalcogenides, such as 2H-MoTe_2_ and 2H-WSe_2_, exhibit efficient CM. Given the similar electronic band structure of 2H-MoSe_2_, it is expected to show comparable CM efficiency. In this study, we establish the occurrence and efficiency of CM in a solution-processed thin film of bulk-like 2H-MoSe_2_. We characterize the dynamics of excitons and free charge carriers by using ultrafast transient optical absorption and terahertz spectroscopy. At higher photon energy the efficiency is comparable to literature results for 2H-MoTe_2_ grown by chemical vapor deposition (CVD) or in bulk crystalline form. At higher photon energies the experimental CM efficiency is reproduced by theoretical modeling. We also observe CM for photon energies below the energetic threshold of twice the band gap, which is most probably due to subgap defect states. Transient optical absorption spectra of 2H-MoSe_2_ exhibit features of trions from which we infer that photoexcitation leads to free charge carriers. We find no signatures of excitons at the indirect band gap. From analysis of the frequency dependence of the terahertz conductivity we infer that scattering of charge carriers in our sample is less than for CVD grown or bulk crystalline 2H-MoTe_2_. Our findings make solution-processed 2H-MoSe_2_ an interesting material for exploitation of CM in photovoltaic devices.

## Introduction

In semiconductors, photoexcitation with photon energies (*E* = *h*ν) exceeding the band gap (*E*_g_) creates energetic electrons and holes, which can be harnessed to generate photocurrent. However, these so-called hot carriers usually quickly lose their excess energy above the band edges through carrier-phonon scattering, typically within subpicosecond time scales. This rapid cooling poses a fundamental barrier to solar light harvesting by single-junction solar cells, it contributes to the Shockley–Queisser (SQ) limit of 33.7% and can also limit the performance of photodiodes.^1−3^

**Scheme 1:**
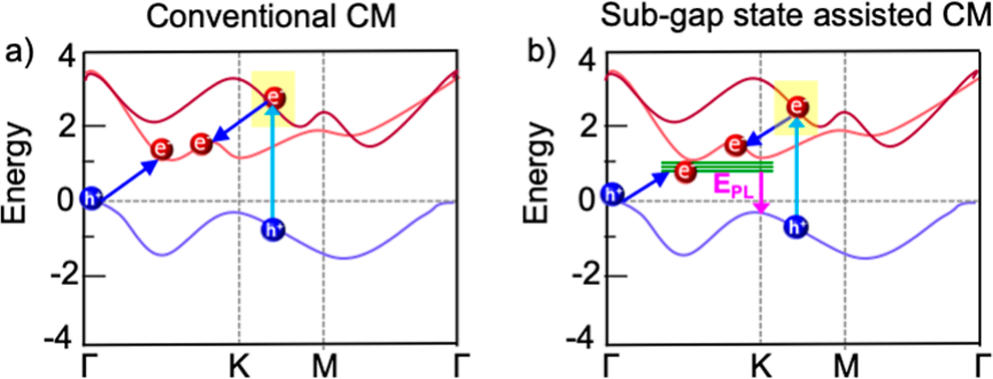
(a) Initial photoexcitation of an electron (light blue arrow) followed by conventional CM. In this case the initially excited electron decays to a lower state by exciting another electron from the valence band to the conduction band, as indicated by the dark blue arrows; (b) Sub-gap defect states (green bars) from which an electron may decay by PL (pink arrow). Sub-gap states can also facilitate CM by an electron with excess energy less than the band gap. As indicated by the dark lue arrows, the upper electron excites an electron from the valence band to a sub-gap state.

One promising strategy to exploit the excess photon energy involves carrier multiplication (CM), where a single high energy photon (*h*ν > 2*E*_g_) generates two or more electron–hole pairs, see Scheme 1a.^3^ This increases the photocurrent, potentially raising the power conversion efficiency of a solar cell to ∼44%.^2,3^ Until several years ago, it was generally thought that CM in nanomaterials with spatially confined electronic states is much more efficient than in bulk semiconductors. This was considered to result from enhanced Coulomb interactions, slow carrier cooling and relaxation of momentum conservation rules for electrons in quantum confined states in nanomaterials, as opposed to delocalized band states in bulk materials.^3^ However, in recent years efficient CM has been found in bulk perovskites and few-layer or bulk two-dimensional (2D) transition metal dichalcogenides (TMDCs).^4−10^

Very efficient CM with a threshold close to twice the band gap has been inferred from transient optical absorption spectroscopy studies on few-layer 2H-MoTe_2_ and 2H-WSe_2_ films grown by chemical vapor deposition (CVD).^6^ In a later study on a CVD grown 2H-MoTe_2_ film a CM threshold of 2.8 times the band gap and an almost staircase-like increase of the quantum yield with photon energy was found from ultrafast terahertz (THz) spectroscopy.^7^ A very recent THz spectroscopy study by Robey et al.^9^ on bulk single-crystal 2H-MoTe_2_ and films grown by CVD yielded CM quantum yields of 1.7 ± 0.3 for a photon energy of 3.1 eV which is close to those from the earlier studies. Interestingly in a preliminary study Robey et al. did not observe any signature of CM for a MoSe_2_ CVD film,^9^ in contrast with expectations CM with exceptionally low energy threshold was recently reported for monolayer MoS_2_ containing intentionally introduced donor states in the band gap.^10^

**Figure 1:**
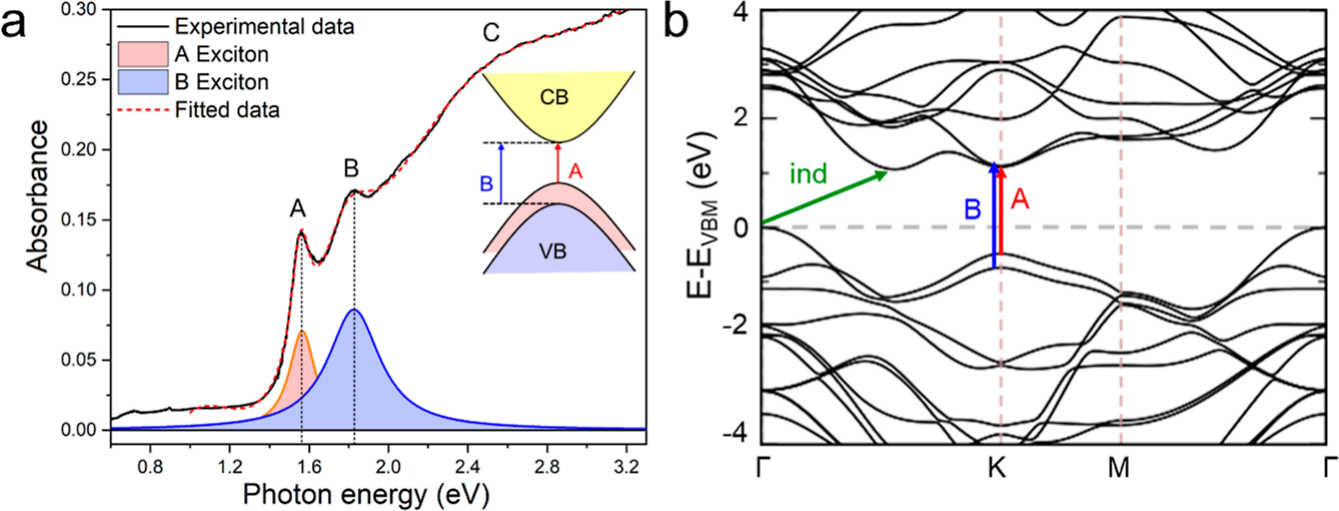
Characterization of the 2H-MoSe_2_ thin film. (a) Ground-state optical absorption spectrum (solid black curve). The dashed red curve represents the fit of eq 1 to the absorption spectrum. Inset: Schematic showing the A and B transitions between the two upper valence band (VB) and the lowest conduction band (CB) states. (b) Electronic band structure obtained from DFT calculations, showing transitions at the direct band gap giving rise to the A exciton (red arrow) and B exciton (blue arrow), as well as the transition at the indirect band gap (green arrow).

According to recent computational modeling, CM in 2H-MoTe_2_ is very efficient due to the large number of possible decay pathways of charge carriers via CM.^11^ The large number of CM pathways in 2H-MoTe_2_ results from a high density of electronic bands with small dispersion as a function of the electron wave vector *k*. Since the electronic energy band diagram of 2H-MoSe_2_ (Figure 1b) is very similar to that of 2H-MoTe_2_,^11^ we expect that the efficiency of CM in these materials is comparable. This is further supported by calculations on MoSe_2_ monolayers that include electron–phonon interactions.^12^

To establish the efficiency of CM in 2H-MoSe_2_ we perform ultrafast transient optical absorption and THz spectroscopy experiments on a thin film. This thin film is produced by solution-processing, which could lead the way to cheap and large scale device fabrication.^13^ We obtain information on the photogeneration and energies of excitons, free charge carriers, trions and biexcitons, as well as scattering and decay dynamics of charge carriers. The CM quantum yields that we determine are evaluated on the basis of our theoretical model reported previously.^11^

## Results and Discussion

### Structural and Optoelectronic Characterization of the 2H-MoSe_2_ Thin Film

An ink of 2D MoSe_2_ flakes was prepared by electrochemical exfoliation of a bulk MoSe_2_ crystal and a thin film is obtained by deposition on a quartz substrate using the Langmuir–Schaefer technique (see Methods). The nanosheet lateral size (*L*) and thickness (*t*) are determined using atomic force microscopy. Figure S1a represents the statistical distribution of *L* and *t* for the nanosheets, with the inset showing a micrograph of two typical MoSe_2_ nanosheets. The average lateral size is ⟨*L*⟩ = 1.78 ± 0.04 μm and the average measured thickness is ⟨*t*⟩ = 12 ± 1 nm. The thickness corresponds to almost 20 MoSe_2_ monolayers,^14^ so that our sample is bulk-like. The Raman spectrum in Figure S1b shows the characteristic peak at ∼245 cm^–1^, originating from the out-of-plane A_1g_ mode in the semiconducting 2H phase. Since the *J*_2_ and *J*_3_ vibrational modes due to metallic 1T phase are not observed, we conclude that our sample consists of the 2H phase.^15,16^ The absence of the 2LA (M) peak (∼300 cm^–1^) due to the longitudinal acoustic phonon at the *M* point in the Brillouin zone implies that the nanosheets are pristine and have minimal defects. We can determine the defect density from the position of the A_1g_ peak which shifts from 245 to 235 cm^–1^ with decreasing interdefect distance (and increasing defect density) from 20 to 0.9 nm, respectively.^16^ Since our A_1g_ peak is located at 245 cm^–1^ the interdefect distance is in the range 4.5–20 nm, which is comparable to similarly prepared MoS_2_ films.^13^ The photoluminescence (PL) spectrum in Figure S1c exhibits a main peak due to emission from the lowest A exciton state and a peak at lower energy with a maximum at 1.24 eV. We assign the latter to subgap defect states (see Scheme 1b) previously observed at this energy for CVD grown MoSe_2_ and attributed to oxygen functional groups.^17^

Figure 1a shows the ground-state optical absorbance spectrum of the MoSe_2_ thin film as a function of photon energy. The peak features denoted as A and B are due to formation of excitons at the direct band gap, which are predominantly composed of an electron at the *K*-point in the lowest conduction band and a hole in the highest or next valence band at the *K*-point, respectively, see inset of Figure 1a.^18−21^Figure 1b shows the electronic band structure of bulk 2H-MoSe_2_ obtained from density functional theory (DFT) calculations (see Methods), where a scissor operator was applied to match the experimental band gap.^22,23^ Bulk 2H-MoSe_2_ has an indirect band gap, indicated by the green arrow. The lowest optically allowed excitations correspond to the momentum direct A and B transitions shown by red and blue arrows, respectively. The broad absorption feature at the high energy side in Figure 1a denoted as C, arises from multiple transitions in the band nesting region where the valence and conduction bands near the Γ point are almost parallel.^18,21,24^ In addition, there is an absorption tail at energies below the A exciton peak, indicating the presence of subgap defect states, which are likely due to oxygen functional groups^17^ as discussed above, or chalcogen vacancies.^10,25,26^

The absorption spectrum, *A*_0_(*E*), can be analyzed by fitting it with the sum of two Lorentzian functions and a fifth-order polynomial function according to^27−29^
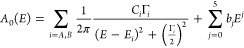
1where the Lorentzian functions account for *A* and *B* exciton transitions, and the polynomial function describes the broad *C* absorption feature at high energy and subgap defect transitions at low energy. The fit of eq 1 (red dashed curve) to the optical absorbance (black solid curve) yields *A* and *B* exciton peak energies at *E*_A_ = 1.565 ± 0.003 eV and *E*_B_ = 1.826 ± 0.002 eV, respectively. The full widths at half-maximum (FWHM) are Γ_A_ = 0.149 ± 0.003 eV and Γ_B_ = 0.364 ± 0.005 eV, which are similar to those for other TMDCs.^29^

### Photogeneration and Decay Dynamics of Excitons, Charge Carriers and Complexes Thereof

**Figure 2:**
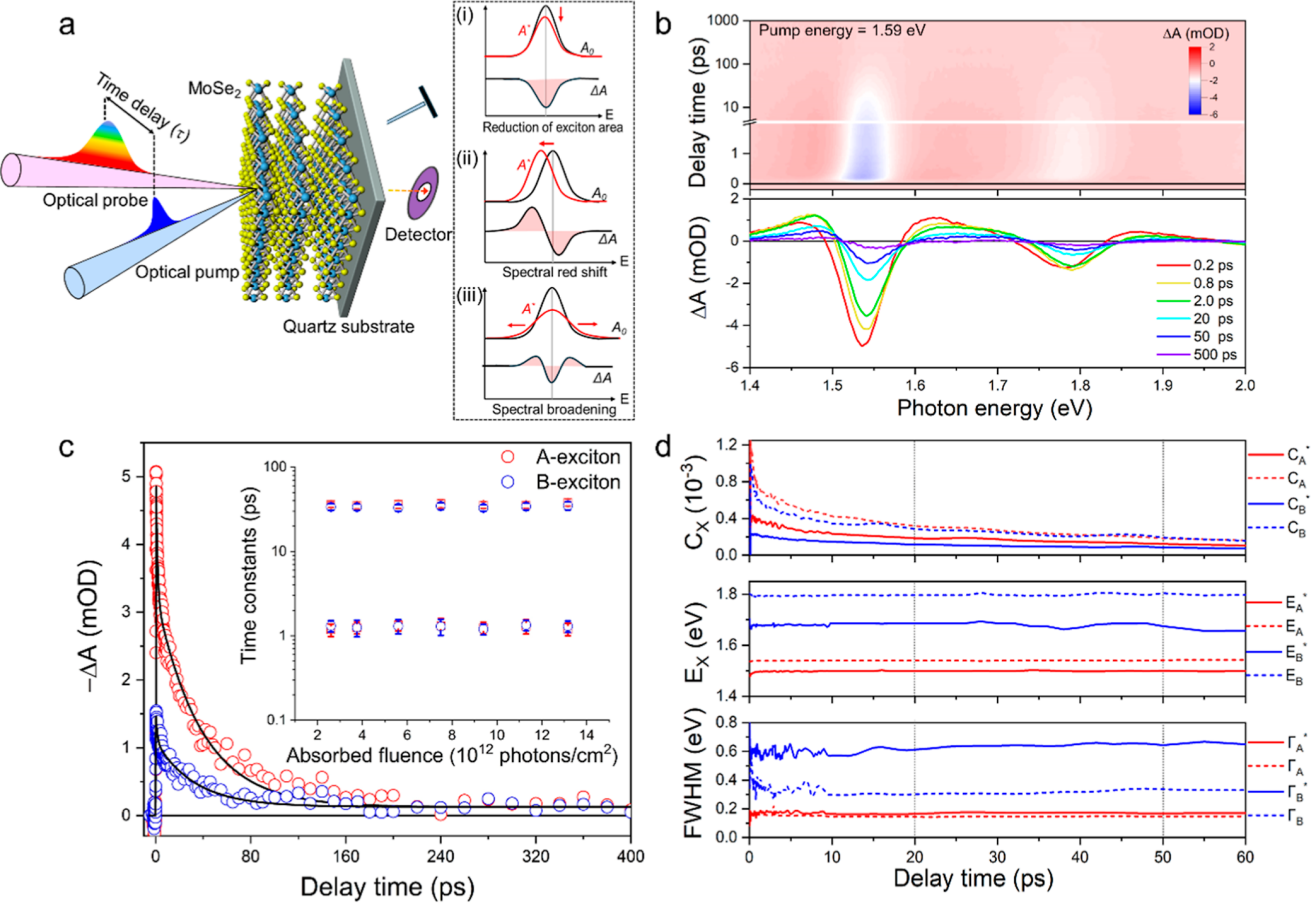
TA spectroscopy at 1.59 eV pump photon energy and absorbed photon fluence of 13.1 × 10^12^ photons/cm^2^. (a) Schematic of TA experiments. Inset: (i–iii) typical transient effects after photoexcitation (b) TA colormap (upper panel) and corresponding TA spectra at the indicated times (lower panel) (c) Decay kinetics of the bleach probed at 1.54 and 1.78 eV below the A (red circles) and B (blue circles) exciton transitions, respectively. The black solid lines represent a biexponential fit and a constant background with decay times shown in the inset also for other pump fluences. (d) Parameters from fits of superpositions of Lorentzian functions (see text) to TA spectra, showing the evolution of amplitudes (*C*_X_, upper panel), energies (*E*_X_, middle panel), and FWHM (Γ_X_, bottom panel) for A (red circles) and B (blue circles) transitions.

We employed transient optical absorption (TA) spectroscopy to investigate the photogeneration and dynamics of charge carriers, excitons, trions and biexcitons (see Methods). As depicted in Figure 2a, an ultrashort laser pulse (180 fs) with tunable photon energy is used to photoexcite electrons from a valence band to an exciton state or a conduction band. After this, a broadband white-light supercontinuum is used to probe the differential change in transmittance, Δ*A*(*E*,τ), of the sample as a function of probe photon energy (*E*) and pump–probe delay time (τ). We determine the TA signal according to , where *I*_on_^probe^ and *I*_off_^probe^ are the transmitted probe light with and without photoexcitation, respectively. The TA signal is equal to the pump-induced change in optical density of the MoSe_2_ film in case the change in reflectivity is negligible.^30^

Figure 2b shows the TA colormap (upper panel) along with TA spectral slices (lower panel) at different pump–probe delay times, following excitation with a pump photon energy of 1.59 eV, which is at the lowest excited state, i.e. the A exciton. The shape of the TA spectra in Figure 2b agrees with those recently reported for multilayer MoSe_2_.^31^ It can be inferred from the band diagram in Figure 1b that the A exciton can relax to lower energy states at the indirect band gap with holes at the top of the valence band near the Γ point and electrons at the conduction band minimum along the Γ–K path, as discussed before for similar materials, including nanoflakes and a bulk crystal of MoS_2_, as well as MoTe_2_ and WSe_2_ films.^6,31−33^ Therefore, the change of the shape of the TA spectrum during the first few picoseconds will at least in part be due to relaxation of A excitons initially produced by the pump pulse to free charges at the indirect band gap. In addition, charge carriers can be trapped at defects. Also, momentum-indirect excitons may be formed at energy below the indirect band gap, as discussed before for several TMDCs.^32,34^ To our knowledge the binding energy of excitons at the indirect band gap in bulk MoSe_2_ is unknown and therefore we cannot estimate to what extent these are populated. Relaxation of A excitons to the above-mentioned optically dark states causes the shape of the TA spectrum to change. The initial change during the first few picoseconds mainly involves the large decay of the optical bleach peaking at 1.54 eV in Figure 2b. This bleach likely stems to a significant extent from the probe pulse-induced photoemission from A excitons. At longer times when A excitons have decayed to the dark states this emission is no longer possible and the TA spectrum is due to (i) reduction of the optical absorption strength, (ii) spectral red-shifts and (iii) broadening, as illustrated in the inset in Figure 2a.^35^ These effects can arise from the following processes.^36^ After relaxation of the initially photoexcited states, there is a population of free/trapped electrons, holes and possibly excitons at the indirect band gap. First, holes at the top of the valence band reduce the optical absorption strength at the A exciton peak, as sketched in panel (i) in Figure 2a. This comes from the fact that the hole state can no longer contribute to the formation of an exciton, which is known as phase-space filling or the “moth-eaten effect”.^36−38^ Second, the probe pulse may produce an electronic excitation near an electron, hole or exciton, leading to formation of a trion or a biexciton, respectively. Trion and biexciton formation occur at a lower oscillator strength and energy than for the original exciton transition,^36,37^ leading to amplitude reduction and red-shift of the absorption peaks, as outlined in panels (i) and (ii) in Figure 2a. In addition, there will be a peak broadening, since the probe photon can produce a trion or biexciton while utilizing the kinetic energy of a charge carrier or exciton produced by the pump pulse, see panel (iii) in Figure 2a. The combination of effects (i–iii) gives rise to the shape of the TA spectra in Figure 2b.

To gain insight into exciton and charge carrier decay mechanisms, we characterized the decay of the TA signals at probe photon energies where the bleach amplitude at 0.2 ps exhibits maxima, which is just below the A and B exciton energies at 1.54 and 1.78 eV, respectively, see Figure 2b. Typical decay traces are shown in Figure 2c, together with fits of two exponentials describing effects of decay of excitons and charge carriers and a constant background due to long-lived species. The decay time constants are independent of absorbed pump photon fluence, see inset of Figure 2c. The absence of an effect of the density of photoexcitations on the decay kinetics indicates a first-order decay due to geminate electron–hole recombination or trapping, rather than higher-order exciton–exciton annihilation as reported for MoSe_2_ multilayer flakes.^31^ A dominant effect of trapping is likely due to presence of more defects in our solution processed thin film sample. We observe a fast decay on a time scale of 1.0–1.5 ps, and a slower decay with a time constant in the range 31–42 ps. The fast component is largest near the A exciton energy, which is likely due to decay of the initially populated A excitons to states at the indirect band gap, while at 1.78 eV the bleach is only due to reduction of oscillator strength and a red-shift of the B exciton peak. The spectral red-shift is evident when comparing the TA spectrum at 5 ps with the first-order derivative of the ground-state absorption spectrum, d*A*_0_/d*E*, see Figure S2. Figure S2 shows that the first-order derivative must be shifted toward lower energy to align it with the TA spectrum, which is typical for a red-shift.^35^

According to the discussion above, the shape of TA spectra is determined by formation of trions and possibly biexcitons by the probe pulse. The latter leads to the appearance of new A* and B* peaks in the absorption spectrum of the sample after the pump pulse, A*(*E*,τ), which exhibit a reduced amplitude, red-shift and broadening as compared to the *A* and *B* peaks in the ground-state spectrum. We describe these effects on A*(*E*,τ) similar to eq 1 by introducing additional Lorentzian functions, *L*_*X* = *A*,B_^*^, for the transitions to trions and possibly biexcitons, and a fifth-order polynomial, *B*_C_^*^, for the background of the excited state. The TA spectrum is then given by Δ*A*(*E*,τ) = A*(*E*,*t*)-*A*(*E*,τ) = [*L*_A_^*^(*E*;*C*_A_^*^, Γ_A_^*^, *E*_A_^*^) + *L*_B_^*^(*E*;*C*_B_^*^, Γ_B_^*^, *E*_B_^*^) + *B*_C_^*^(*E*;*b*_j_^*^)] – [*L*_*A*_(*E*;*C*_*A*_, Γ_*A*_, *E*_*A*_) + *L*_*B*_(*E*;*C*_*B*_, Γ_*B*_, *E*_*B*_) + *B*_*C*_(*E*;*b*_*j*_)]. The fit parameters *C*_X_^*^ (*C*_*X*_), *E*_X_^*^ (*E*_*X*_), Γ_X_^*^ (Γ_*X*_), and *b*_j_^*^ (*b*_*j*_) represent the magnitude, peak energy and line width of the Lorentzian functions and the fifth-order polynomial in the spectrum of the excited (nonexcited) sample. The fits excellently reproduce the TA spectra, see representative results in Figure S3. Figure 2d shows the fitted parameters for pump photon energy of 1.59 eV (near the A exciton peak) as a function of delay time, τ, after the pump pulse. The exciton peak energies and line widths for the nonexcited sample are similar to those obtained from the fit of eq 1 to the ground-state absorption spectrum in Figure 1a, as expected. On short times the probe-induced photoemission from A excitons leads to a bleach that is taken into account by the magnitude of *C*_*A*_. This fast initial decay of *C*_*A*_ in Figure 2d reflects the decay of *A* excitons into charge carriers by transfer of electrons to the indirect band gap or traps, or to excitons at the indirect band gap. The rapid initial decay of *C*_*B*_ reflects a strong initial bleach of the *B* exciton peak. The bleach of the *B* exciton peak due to the presence of *A* excitons can result from a reduction of the cross section for photogeneration of *B* excitons by phase-space filling (“moth-eaten effect”).^36−38^ This would imply that Bloch states contributing to the wave function of the *A* exciton are no longer available for formation of a *B* exciton.

After decay of the *A* excitons, the amplitudes *C*_*X*_ and *C*_X_^*^ are both proportional to the density of charge carriers and possibly excitons at the indirect band gap. Further, the parameter *C*_*X*_ is proportional to the cross section (oscillator strength) of photogeneration of an exciton (*X* = *A*, *B*) by excitation from the ground state. The value of *C*_X_^*^ scales with the cross section of formation of an *X* exciton by the probe pulse near a charge carrier or an exciton at the indirect band gap formed by the pump pulse. Hence, the magnitude of *C*_X_^*^ is determined by the cross sections for formation of trions and biexcitons scaled with the density of charge carriers and excitons left after the pump pulse, respectively. On longer times the decay kinetics of *C*_*X*_ and *C*_X_^*^ are similar and reflect the recombination of charge carriers and excitons to the ground state. Interestingly, the ratio *C*_*X*_/*C*_X_^*^ > 1, which reflects that the cross sections of photogeneration of trions or biexcitons are smaller than for excitons, in agreement with theory.^36,37^ The difference between the exciton energy *E*_*A*_ and *E*_A_^*^ is ∼30 meV, which is close to trion binding energies reported for monolayer MoSe_2_^19,39,40^ and smaller than the biexciton binding energy of 57 meV for bilayer MoSe_2_.^41^ This suggests that the A* peak is predominantly due to formation of trions, while the contribution of biexcitons is negligible. The latter implies that photoexcitation leads mainly to charge carrier generation and to a much smaller extent to excitons at the indirect band gap. Figure 2d shows that the energy difference *E*_*B*_ – *E*_B_^*^ is ∼100 meV. This means that the trion binding energy involving B excitons is higher than for A excitons. The FWHM value of the A* peak is ∼26 meV higher than that of the A peak, see Figure 2d. The broader A* peak will be due to the shorter lifetime of trions compared to excitons, resulting from Auger recombination or dissociation into an exciton and a free charge carrier as additional decay channels of trions. In addition, the (thermal) energy distribution of charge carriers available for trion formation leads to a low-energy tail. The FWHM of the B* peak is about 300 meV larger than that of the *B* peak. The FWHM of the B* peak is larger than that of the *B* peak due to the same factors as for the A* and *A* peaks, and the difference will be further enhanced by the shorter lifetime of B* trions due to relaxation of B* trions to A* trions. We do not know the exact reason for the much larger the difference between the FWHM of the B* and *B* peaks as compared to the A* and *A* peaks.

In summary, from analysis of the TA spectra discussed above we infer that *A* excitons relax on a time scale well within 10 ps, with the products predominantly being charge carriers rather than excitons at the indirect band gap.

### Determination of Carrier Multiplication Quantum Yield by TA Spectroscopy

**Figure 3:**
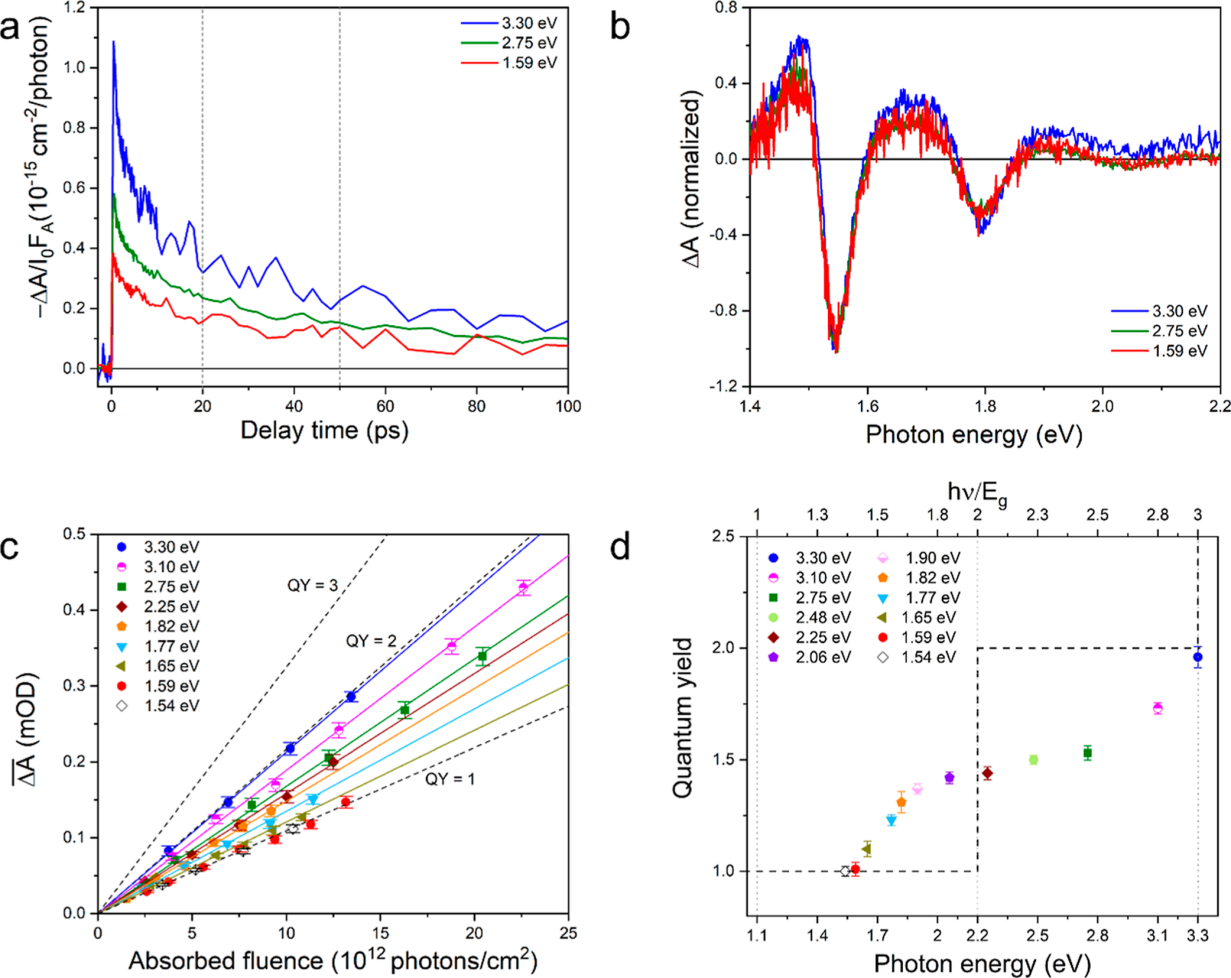
Investigation of CM by TA spectroscopy. (a) Effect of pump photon energy on the decay kinetics of the TA signal normalized to absorbed pump fluence [in the range (3.7–4.1) × 10^12^ photons/cm^2^], recorded at 1.54 eV where the bleach is highest. The vertical gray dotted lines indicate the 20–50 ps time interval used to determine the CM quantum yield. (b) TA spectra normalized at 1.54 eV and averaged over delay times in the range 20–50 ps for different pump photon energies. (c) Integrated absolute TA signal, , as a function of absorbed photon fluence for different pump photon energies. The solid lines are linear fits to the experimental data, with their slope representing the quantum yield. Error bars represent the standard deviations. (d) Quantum yields plotted as a function of photon energy (bottom axis) and band gap multiple (top axis). The dashed step-like black curve illustrates the scenario of ideal CM.

Having characterized the origin and decay of the features in the TA spectrum we turn to investigation of CM. To this end we determine the variation of the quantum yield of photoexcited electrons and holes as a function of the energy of the pump photon. Figure 3a shows the time-dependence of the magnitude of the TA signal at a probe photon energy of 1.54 eV where the bleach is highest (see Figure 3b), normalized to the absorbed pump photon fluence *I*_0_*F*_*A*_, with *I*_0_ the incident number of pump photons per unit area and *F*_*A*_ the fraction absorbed. The magnitude of the TA signal in Figure 3a increases with photon energy with the effect being largest during the first 10 ps. The relatively strong bleach near 1.54 eV on short time is further illustrated in the TA spectra in Figure S4. For photoexcitation at 1.59 eV the initial bleach at 1.54 eV will be in part due to probe-induced photoemission from the A exciton, as discussed above. At higher pump photon energy the large initial bleach can be due to population of electron or hole states at the *K*-point in the conduction and valence bands (see Figure 1), which reduces the cross section for A exciton formation by phase-space filling,^36−38^ and in addition by a decrease of the cross section by the Stark effect induced by hot charge carriers.^28^Figure 3b shows the TA spectra averaged over delay times in the range 20–50 ps. The spectra are effectively independent of pump photon energy, as expected, since these times are much longer than previously reported charge carrier relaxation times in TMDCs of the order of picoseconds.^6,28,42,43^ After 20 ps the increase of Δ*A*/*I*_0_*F*_*A*_ with pump photon energy in Figure 3a thus directly reflects a higher quantum yield of charge carriers due to CM.

As discussed above, charge carriers cause a change of the absorption spectrum, or equivalently a change of the absorption cross section Δσ(*E*) at probe photon energy *E*. In that case the TA signal at time τ after the pump pulse is Δ*A*(*E*,τ) = Φ*I*_0_*F*_*A*_*s*(τ)Δσ(*E*)/ln(10), with Φ the initial quantum yield for charge carrier photogeneration and *s*(τ) the survival fraction of electron–hole pairs.^3,4^ The variation of the slope of Δ*A*(*E*,τ) versus the absorbed photon fluence, *I*_0_*F*_*A*_, with photoexcitation energy thus directly reflects the change of Φ. To enhance the signal-to-noise ratio we consider the integrated absolute TA signal, , which is the sum of the absolute values of Δ*A*(*E*,τ) for all energies in the TA spectra averaged over delay times τ in the interval 20–50 ps, similar to our study on CM in a perovskite material.^3,4^

Figure 3c shows magnitude of  as a function of absorbed photon fluence for different pump photon energies. The linear increase of  with fluence shows that effects of higher-order recombination processes of electron–hole pairs are negligible. This is further illustrated by the absence of a fluence effect on the decay kinetics of the TA signals in Figure S5. Linear fits to the fluence dependence of  provide a slope for each pump photon energy, which is proportional to the quantum yield Φ. Hence, the rise of the slope with pump photon energy reflects the increase of the quantum yield. To within the experimental uncertainty the slopes are the same for the two lowest pump photon energies of 1.54 and 1.59 eV. Assuming a unit quantum yield for the lowest energy, we obtain the quantum yield as a function of photon energy shown in Figure 3d. The quantum yield starts to exceed 1.0 at a photon energy of *h*ν = 1.65 eV, then it is close to 1.5 for *h*ν = (2.0–2.8) eV and reaches a value of almost 2.0 at *h*ν = 3.3 eV. The top axis shows the photon energy in terms of the band gap multiple, *h*ν/*E*_g_, with *E*_g_ = 1.1 eV the experimental band gap of MoSe_2_ from literature.^22,23^ The quantum yield starts to exceed 1.0 for photon energy below the “ideal” threshold energy of twice the band gap. According to theory,^12^ CM in TMDCs can occur at photon energy below twice the band gap, due to electron–phonon assisted transitions and involvement of subgap states due to oxygen functional groups^17^ as discussed above, or chalcogen vacancies.^10,25,26^ These effects can explain the optical absorbance in Figure 1a below the A exciton peak down to 0.6 eV, and consequently a CM threshold near 1.6 eV, which can be understood as follows. Scheme 1b shows a CM process with involvement of a subgap state. The observed PL peak at 1.24 eV (see Figure S1c) is likely due to decay of an electron from a subgap state at 1.24 eV above the hole state at the top of the valence band at the *K*-point. According to the band diagrams in Figure 1b and Scheme 1b, the energy of the subgap state is then 0.74 eV above the top of the valence band at the Γ-point and below the bottom of the conduction band at 1.09 eV (see Methods). The possibility of CM by exciting an electron from the top of the valence band at the Γ-point to this subgap state reduces the photon energy at which CM becomes energetically allowed to 1.48 eV. This is even below the lowest photon energy of 1.65 eV at which we observe CM. Hence, oxygen functional groups and/or other defects can reduce the onset energy of CM to a value below twice the indirect band gap (i.e., below 2.18 eV).

The quantum yields for solution-processed 2H-MoSe_2_ in Figure 3d are comparable to or exceeding previous results for CVD grown few-layer or bulk crystalline 2H-MoTe_2_ and 2H-WSe_2_ obtained from pump–probe spectroscopy with optical or terahertz conductivity detection.^6,7,9^ This makes solution-processed 2H-MoSe_2_ another interesting candidate for applications in photovoltaic devices that exploit CM.

### Investigating Carrier Multiplication and Charge Dynamics by Terahertz Spectroscopy

**Figure 4:**
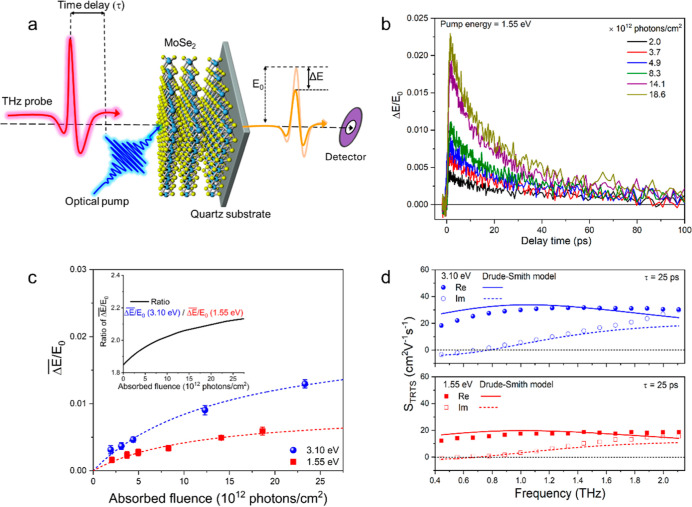
Investigation of CM by THz spectroscopy. (a) Schematic depiction of optical pump-THz probe experiments. (b) Pump fluence-dependent OPTP signals, Δ*E*(τ)/*E*_0_, upon excitation at 1.55 eV. (c) OPTP signals averaged in the interval 20–50 ps as a function of absorbed photon fluence, following photoexcitation at 1.55 eV (red squares) or 3.10 eV (blue circles). The dotted lines represent fits of the saturation function , and the inset shows the ratio of the fits for 3.10 and 1.55 eV. (d) Frequency-dependent complex photoconductivity signals obtained from TRTS measurements at a delay time τ = 25 ps after photoexcitation at 1.55 or 3.10 eV and low absorbed photon density of (2.0 ± 0.1) × 10^12^ photons/cm^2^. The solid and dotted lines represent fits of the Drude-Smith model to the real and imaginary conductivity components, respectively.

We also used terahertz (THz) spectroscopy (see Methods) to study CM and charge carrier dynamics,^44−46^ similar to previous studies on few-layer and bulk 2H-MoTe_2_.^7,9^ The THz technique is illustrated in Figure 4a. First, we carried out optical-pump terahertz probe (OPTP) measurements, where a pump laser pulse photoexcites electrons and the reduction, Δ*E*(τ), of the maximum, *E*_0_, of the THz waveform is measured as a function of delay time τ after the pump pulse. The ratio Δ*E*(τ)/*E*_0_ is proportional to the densities of electrons and holes weighted by their respective real mobility components averaged over the frequencies in the THz waveform, see Methods eq 3. Figure 4b shows Δ*E*(τ)/*E*_0_ measured after photoexcitation at 1.55 eV. We observe an instantaneous signal rise due to charge carrier generation, followed by decay on a time scale of tens of picoseconds. The decay kinetics is only weakly dependent on pump photon fluence and energy, see also Figures S6–S8. This implies that charge carriers decay predominantly by first-order recombination or trapping at defects rather than via higher-order (Auger) recombination. In addition, Figure S9 shows that the decay kinetics of the THz (OPTP) and TA bleach signals (probed at 1.54 eV) are similar after ∼20 ps, both for low and high pump photon energies. This implies that the initially photogenerated excitons or energetic charge carriers have relaxed to the same electron and hole states at the band edges or in traps.

**Figure 5:**
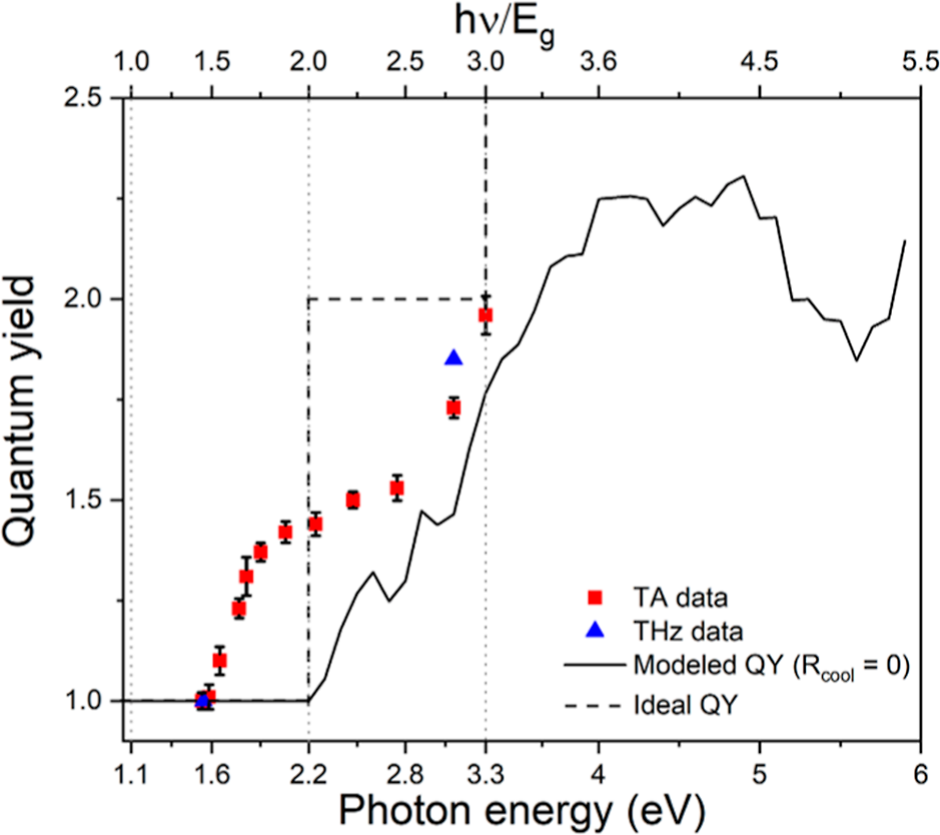
Calculated quantum yield (black solid line) in the absence of carrier cooling, along with experimental data from TA spectroscopy (red squares) and THz conductivity measurements (blue triangles), as a function of photon energy (bottom axis) and band gap multiple (top axis). The black dashed line represents the case of ideal stair-like CM.

We determine the efficiency of CM from the OPTP signal, , averaged over times τ in the interval 20–50 ps, as we did for the TA signals in Figure 3c. Figure 4c shows such averaged OPTP signals as a function of absorbed pump fluence for a photon energy of 1.55 eV (below the CM threshold, see Figure 3c) and at 3.10 eV which is well above twice the band gap. The OPTP signals increase sublinearly with fluence, which likely results from reduction of the charge carrier mobility with fluence due to enhanced carrier–carrier scattering at higher carrier density. We fit the saturation function  to the data in Figure 4c, where α is a constant, *I* the absorbed pump fluence and *I*_s_ the saturation fluence.^9,47^ This yields α = 0.009 ± 0.001 and *I*_s_ = (12 ± 3) × 10^12^ cm^–2^ for a pump energy of 1.55 eV and α = 0.021 ± 0.002 and *I*_*s*_= (15 ± 3) × 10^12^ cm^–2^ for 3.10 eV. The ratio of the fits in the inset of Figure 4c provides a measure of the quantum yield at photon energy of 3.10 eV, since at 1.55 eV it is considered to be equal to 1, see Figure 3c. To minimize nonlinear effects at higher charge carrier density we take the ratio of the fitted values at low fluence and obtain a quantum yield of 1.85 for a photon energy of 3.10 eV. This value is close to the TA results, see Figure 5, and is similar to results for 2H-MoTe_2_.^7,9^ Note that the saturation fluences at 1.55 and 3.10 eV are similar, despite the initially higher charge carrier density at 3.10 eV due to a larger absorbance (see Figure 1) and occurrence of CM. Apparently, after 25 ps the spatial distribution of charge carriers has become similar due to their random motion through the sample.

The frequency dependent terahertz conductivity signals *S*_TRTS_(ω,τ) obtained at delay time τ = 25 ps after photoexcitation at 1.55 and 3.10 eV are shown in Figure 4d. The complex valued *S*_TRTS_(ω,τ) is the sum of the mobilities of electrons and holes, μ_*e*,*h*_(ω), weighted by their time-dependent quantum yield, Φ_*e*,*h*_(τ), and possibly a contribution of excitons, see Methods eq 4. The ratio of both the real and imaginary conductivity signals for 3.10 and 1.55 eV photon energy are similar to that of the OPTP results in the inset in Figure 4c. This corroborates the occurrence of CM at 3.10 eV.

To obtain insight into charge carrier scattering processes we fit the frequency dependence of the THz signal by the Drude-Smith model that can account for anisotropic scattering on defects and is given by^48−51^
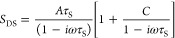
2

In eq 2, τ_S_ is the charge scattering time, the parameter *C* brings into account the anisotropy of scattering at defects and ranges from *C* = 0 for isotropic scattering in random directions to *C* = −1 for full backscattering (180 deg). The amplitude *A* is proportional to the ratio of the quantum yield of charge carriers and their effective mass. As can be seen in Figure 4d, the Drude-Smith model reproduces the data reasonably well with fit parameters *A* = 0.18 ± 0.02, τ_S_ = 116 ± 14 fs and *C* = −0.63 ± 0.07 for photon energy of 1.55 eV and = 0.31 ± 0.03, τ_S_ = 118 ± 12 fs and *C* = −0.67 ± 0.06 for 3.10 eV. The similar values of the scattering parameters τ_S_ and *C* for both pump photon energies substantiate that charges have fully relaxed after 25 ps. The scattering times found for our solution-processed 2H-MoSe_2_ film are ∼30% longer than for bulk crystalline 2H-MoTe_2_,^9^ while the *C* values are slightly less negative. This suggests that the charge mobility in the present solution-processed sample can exceed that for crystalline 2H-MoTe_2_. Our finding of nonzero negative *C* values implies that charges undergo preferential backscattering at defects rather than isotropic scattering, as would be described by the Drude model (i.e., *C* = 0). The charge carrier quantum yield at 3.10 eV can be calculated as the ratio of the parameter *A* for 3.10 and 1.55 eV, which is equal to 1.72 ± 0.34, in agreement with the results from our TA and OPTP experiments, see Figures 3 and 4.

### Theoretical Modeling

We calculated the CM efficiency using our theoretical model applied to 2H-MoTe_2_ previously.^11^ In the Methods section we specify details of the calculation of the electronic band structure of 2H-MoSe_2_ and the quantum yield for charge carrier generation as a function of photon energy, using eq 5. We neglected cooling of charge carriers by phonon emission and effects of trapping of charge carriers at defects. Figure 5 depicts the calculated quantum yield along with the experimental data from TA and THz spectroscopy. At higher photon energies the calculated quantum yield is close to the experimental data. This suggests that CM outcompetes carrier cooling due to weak electron–phonon coupling, as found for 2H-MoTe_2_.^7^ Note that the model cannot reproduce our observation of CM below twice the band gap of MoSe_2_. To describe the latter, effects of subgap defects on CM must be included into the model.

Figure S10 shows the total summed average density of CM pathways, , along with individual contributions from electrons () and holes (), as a function of band gap multiple (*h*ν/*E*_g_). Interestingly, we find that  increases rapidly with photon energy with a more pronounced contribution from holes for photon energies up to *h*ν/*E*_g_ ≅ 4.5, followed by a dominant contribution of electrons at higher energy.

## Conclusions

We have observed efficient CM in solution-processed 2H-MoSe_2_ with energy threshold below twice the band gap due to subgap defect states. At higher photon energy the efficiency is consistent with previous results for CVD grown or bulk crystalline 2H-MoTe_2_ and can be reproduced by our theoretical model. Photoexcitation of 2H-MoSe_2_ leads to charge carriers and signatures of excitons at the indirect band gap are not observed. We determine trion binding energies ∼30 meV for *A* excitons and ∼100 meV for *B* excitons. Scattering of charge carriers in our sample is less than for CVD grown or bulk crystalline 2H-MoTe_2_. According to our results, solution-processed 2H-MoSe_2_ is a promising material for exploitation of CM in photovoltaic devices. Future studies on controlling defect states and photovoltaic device performance of TMDC materials will be of interest.

## Methods

### Electrochemical Exfoliation of a Bulk MoSe_2_ Crystal

A nanosheet ink is prepared from a bulk crystal of 2D MoSe_2_ using electrochemical exfoliation, as described elsewhere.^13^ An electrochemical setup is used to intercalate a thin piece (0.1 × 1 × 1 mm) of MoSe_2_, which is employed as the cathode, while a platinum foil (Alfa Aesar) is used as the anode. The electrolyte solution is prepared by dissolving tetrapropylammonium bromide (Sigma-Aldrich, 5 mg/mL) in approximately 50 mL of propylene carbonate. A potential of 8 V is applied for 30 min between the electrodes to intercalate the MoSe_2_ crystal with TPA^+^ cations, as indicated by more than a 2-fold expansion of the original crystal volume. The intercalated material is washed with dimethylformamide (DMF) to remove any residual bromide, TPA^+^ cations and propylene carbonate on the surface of the crystal. Then, the crystal is bath-sonicated in a 1 mg/mL poly(vinylpyrrolidone) (molecular weight ∼40,000) solution in DMF for 5 min, followed by centrifugation (Hettich Mikro 220, 1195-A, radius 87 mm) at 500 rpm (24 g) for 20 min to remove unexfoliated crystals. The dispersion is size-selected by centrifuging the supernatant (top 90%) at 1000 rpm (97 g) for 1 h and the sediment is collected. The sediment is then diluted with 2 mL of DMF and centrifuged at 10,000 rpm (9744 g) for 1 h to remove residual PVP and this step is repeated twice. To remove DMF, the sediment is diluted in 0.5 mL of isopropanol (IPA) followed by centrifugation at 10,000 rpm (9744 g) and then the sediment is collected. In the final step, the sediment is redispersed in ∼0.5 mL of IPA (concentration ∼2.5 g/L) to prepare nanosheet inks, which is then used to fabricate 2H-MoSe_2_ thin film.

### Thin Film of 2H-MoSe_2_ (Langmuir–Schaefer Deposition)

A thin film of 2H-MoSe_2_ with a thickness of 28 nm is prepared on a quartz substrate (2.5 × 1.1 cm, Esco Optics) from a nanosheet ink using Langmuir–Schaefer-type deposition. A beaker (250 mL) is filled with deionized water until the substrate on the substrate holder is completely submerged. Then, distilled *n*-hexane (∼2 mL) is added to establish the liquid/liquid interface. Using a Pasteur pipet, the nanosheet inks are carefully injected into the interface until a uniform film formed. The substrate is then lifted through the interface to transfer the nanosheet layer and the wet substrate is allowed to air-dry at room temperature. Finally, the dry film is annealed at 120 °C for 1 h under an argon atmosphere to remove any remaining water. The deposition, followed by annealing is repeated a second time to complete the film.

### Optical Absorption Spectroscopy

The ground-state optical absorption spectrum of the MoSe_2_ thin film is recorded using a double beam PerkinElmer Lambda 1050 UV–vis spectrometer. The sample is measured inside an integrating sphere and an empty quartz substrate is measured separately for background correction.

### Raman and Photoluminescence Spectroscopy

The MoSe_2_ ink is drop cast onto a Si/SiO_2_ substrate, then heated to 120 °C for annealing in a glovebox filled with nitrogen. A 100× objective on a Reinshaw Raman spectrometer operating at 532 nm is utilized to obtain spectra. An incidence power of approximately 1 mW was applied to reduce the possibility of thermal damage.

### Atomic Force Microscopy

AFM measurements are carried out with a Bruker Multimode 8 microscope, which analyses lateral nanosheet size and thickness. The ink is drop-cast onto silicon/silicon dioxide (Si/SiO_2_) substrates after being diluted 1:100 with IPA. After diluting, the sample is annealed for 30 min at 120 °C to remove any remaining solvent. The sample is systematically scanned using OLTESPA R3 cantilevers in the ScanAsyst mode. The nanosheet lateral size, *L*, is calculated as the square root of the product of the nanosheets length and width.

### Transient Optical Absorption (TA) Spectroscopy

TA measurements are performed on a thin film of MoSe_2_ loaded inside an airtight brass holder inside a nitrogen-purged glovebox. Detailed experimental setup is described previously.^52^ Briefly, a Yb-KGW oscillator (Light Conversion, Pharos SP) is used to generate 180 fs laser pulses at 1028 nm with a repetition rate of 5 kHz. Tunable pump pulses (330–1330 nm) are obtained by nonlinear frequency mixing of the fundamental beam through an Optical Parametric Amplifier equipped with a second harmonic module (Light Conversion, Orpheus). A small portion of the fundamental beam is directed to a sapphire crystal to produce a broadband probe spectrum (480–1600 nm) by supercontinuum generation. The pump beam is transmitted through a mechanical chopper operating at 2.5 kHz, allowing one in every two pump pulses to pass. The pump and probe beam overlapped at the sample with relatively small angle of ∼8° and the delay time between pump and probe is controlled by an automated delay stage. After passing through the sample, the pump beam is dumped, while the probe beam is collected by a detector (Ultrafast Systems, Helios). During the measurements, the polarizations of the pump and probe beams are set to be orthogonal to reduce the influence of pump scattering at the detector. All the TA data are corrected for probe-chirp via a polynomial correction to the coherent artifact. We determined the pump fluence by measuring the beam profile with a camera beam profiler (Thorlabs, BC106-VIS), as shown by representative images in Figure S11.

### Terahertz (THz) Spectroscopy

The THz photoconductivity dynamics is determined from optical-pump terahertz-probe (OPTP) and time-resolved THz spectroscopy (TRTS) measurements, as described previously.^44,46^ Our THz spectroscopy setup is based on an amplified Ti:sapphire laser system (Coherent, Libra), producing 60 fs pulses with a center wavelength of 800 nm and a repetition rate of 1.4 kHz. The output of the amplifier is split into three parts for (1) photoexcitation (pump) of the sample, (2) THz generation, and (3) THz detection. The first part of the beam can be optically converted to a pump wavelength of 400 nm (photon energy 3.1 eV) in a BBO crystal via frequency doubling. The second part is used for generation of a THz waveform with a duration of ∼1 ps in a ZnTe crystal via optical rectification. The third part is used for detection of the THz waveform after transmission through the sample, which occurs in another ZnTe crystal via electro-optic sampling. Time delays between the photoexcitation pump pulse and the THz detection pulse (τ) and between the THz generation and detection pulse (*t*) are controlled by mechanical delay stages. To avoid THz absorption by air the measurements are performed in a closed box under N_2_ atmosphere.

We measure the time dependent transmitted THz waveform of the sample without photoexcitation, *E*^off^(*t*), by so-called THz time-domain spectroscopy (THz-TDS).

#### OPTP Measurements

During the OPTP measurements, we photoexcited the sample with chopped pump laser pulses to obtain the difference, Δ*E*(τ), of the maximum of the transmitted THz waveform at a delay τ after the pump pulse. Hence, Δ*E*(τ) = *E*^off^(*t*_max_) – *E*^on^(*t*_max_,τ), where *t*_max_ is the time at which the THz waveform is maximum without photoexcitation of the sample. In the main text we write *E*^off^(*t*_max_) ≡ *E*_0_ for brevity. From these measurements we can determine the real part of the photoconductivity averaged over the frequencies in the THz waveform, provided the phase shift of the THz waveform due to the imaginary photoconductivity is small.^49,53^ The sum of the mobility of free electrons and holes, (μ_R*,e,h*_) and the exciton (EX) response (μ_R,EX_), weighted by their time-dependent quantum yield, Φ(*t*), at time τ after the pump pulse can be obtained according to^51,54^
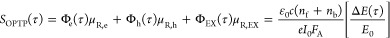
3

In the equation above, *I*_0_*F*_A_ is photoexcitation density per unit area, ε_0_ is the vacuum permittivity, *c* is the speed of light, while *n*_f_ and *n*_b_ are the refractive indices of the media in front and back of the sample, respectively. For the film of MoSe_2_ on a quartz substrate we use *n*_f_ = 1 (for N_2_) and *n*_b_ = 2 (for the quartz substrate).^55^ The real part of the mobility of free charge carriers arises from the in-phase motion of the carriers with the THz electric field, whereas the real part of exciton mobility is related to the absorption of THz radiation by excitons.^49,53^

#### TRTS Measurements

We measure the change of the THz waveform at different delay times, τ, after photoexcitation of the sample by chopping the pump laser pulse and scanning the delay time, *t*, of the THz generation pulse. Together with *E*^off^(*t*) from the THz–TDS measurement we obtained the frequency dependent THz conductivity according to^51,54^
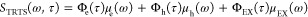

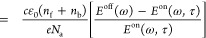
4with *E*^off^(ω) and *E*^on^(ω,τ) the Fourier transforms of the THz waveforms at radian frequency ω = 2π*f*. Note that mobilities in the above equation are complex valued.

### Electronic Band Structure Calculations

Electronic band structures are obtained from density functional theory (DFT) calculations. The calculations are done with pseudopotentials and a generalized gradient approximation (GGA) exchange–correlation functional using the ABINIT package.^56^ For the calculations on MoSe_2_, we used a hexagonal structure with lattice constants *a* = 3.26 Å and *c* = 12.83 Å, as obtained from literature.^14^ Additionally, a 14 × 14 × 7 *k*-point grid with an energy cutoff of 40 hartree (∼1088 eV) is employed. The indirect and direct band gaps from the DFT calculations are 0.86 and 1.48 eV, respectively. A scissor operator of 0.23 eV is applied to adjust the bands, matching the experimental indirect band gap of 1.09 eV.^22^

### Modeling Carrier Multiplication

The quantum yield is modeled from the DFT calculations as described in our previous article.^11^ Briefly, from iteratively comparing sets of four electronic states, which represent the initial and final states of two scattering electrons, and counting the number of sets that meet the conditions for CM, we obtain the number of CM pathways (*N*_CM_) for each initial excited state. This quantity can be related to the probability of CM (*P*_CM_), which then can be used to calculate the quantum yield using the expression
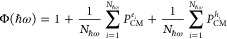
5where *N*_ℏω_ are the number of allowed optical transitions with a photon that carries ℏω energy, and  and  are the probability of CM for the excited electron (e) and hole (h) of optical transition *i*. In the framework of the previous article,^11^*P*_CM_ is a function of *N*_CM_, a rate constant (*F*_CM_), and the rate of carrier cooling (*R*_cool_), which competes with CM. In this article, carrier cooling is neglected (*R*_cool_ = 0) and the modeled quantum yield therefore represents an upper limit. In this case, the probability of CM for optical transition *i* (P_CM_^*i*^) is defined as the fraction of optical transitions at a given photon energy that leads to populating a state which has at least one carrier multiplication pathway (*N*_CM_^*i*^ > 0), i.e.
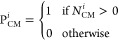


## Data Availability

The authors declare that the data supporting the findings of this study are available within the paper and its Supporting Information files. Data is also available from the corresponding author upon reasonable request.

## Supporting Information Available

Raman spectra, AFM micrograph and statistics, PL spectrum, comparison of TA spectrum and derivative of steady-state absorption, fitted TA spectra, additional TA spectra and kinetics, OPTP data at 3.10 eV pump photon energy, THz decay kinetics for different pump fluences and photon energies, comparison of TA and THz conductivity decay kinetics, calculated CM pathway densities for electrons, holes and their sum, and laser beam profile images (PDF)
