# Supplementary material for: Carrier Multiplication and Photoexcited Many-Body States in Solution-Processed 2H-MoSe2
Source: ACS Nano. 2025 Mar 6;19(10):10347–58. doi: 10.1021/acsnano.4c18254 (PMC11924332; doi:10.1021/acsnano.4c18254)
Supplement: Supplementary file 1 — nn4c18254_si_001.pdf [file nn4c18254_si_001.pdf]

Supporting Information

Carrier Multiplication and Photoexcited Many-Body States in Solution-Processed 2H-$MoSe_2$

Goutam Ghosh[1*], Tian Carey[2], Stevie Furxhiu[1], Sven Weerdenburg[1], Nisha Singh,[1] Marco van der Laan[3], Susan E. Branchett[4], Sophie Jaspers[1], John W. Suijkerbuijk[1], Fedor Lipilin,[5] Zdeněk Sofer,[5] Jonathan N. Coleman[2], Peter Schall[3], Laurens D.A. Siebbeles[1*]

[1]*Chemical Engineering Department, Delft University of Technology, Van der Maasweg 9, 2629 HZ Delft, The Netherlands*

[2]*School of Physics, CRANN & AMBER Research Centres, Trinity College Dublin, Dublin 2, Ireland*

[3]*Institute of Physics, University of Amsterdam, Amsterdam 1098 XH, The Netherlands*

[4]*ICT Innovation, Delft University of Technology, Landbergstraat 15, 2628 CE, Delft, The Netherlands*

[5]*Department of Inorganic Chemistry, University of Chemistry and Technology Prague, Technická 5, Prague 6, 166 28, Czech Republic*

*Correspondence should be addressed to G.G. and L.D.A.S

Emails: G.Ghosh@tudelft.nl, l.d.a.siebbeles@tudelft.nl

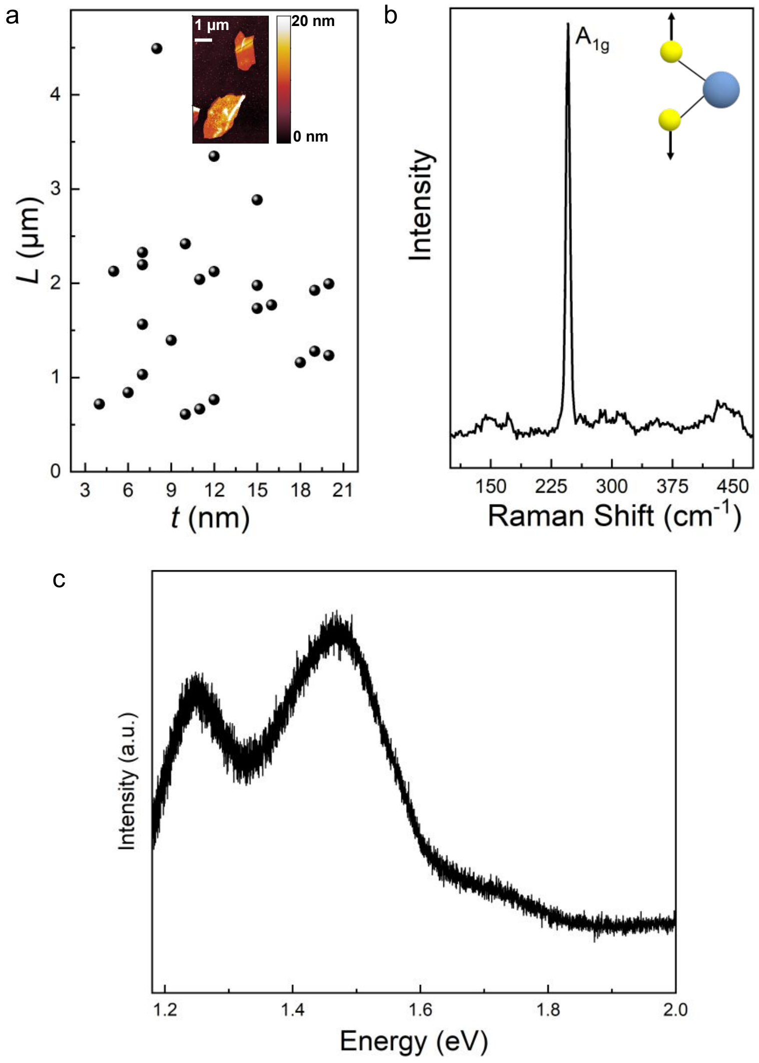

Figure S1. (a) Statistical distribution of nanosheet lateral size (*L*) as a function of measured thickness (*t*) from AFM, where each data point represents a single nanosheet. Inset: Two typical AFM micrographs showing $MoSe_2$ nanosheets drop-cast onto a Si/SiO_2 substrate. (b) Raman spectrum of semiconducting 2H-$MoSe_2$ upon 532 nm excitation. Inset: The schematic of out-of-plane vibrational mode corresponding to A_{1g} mode, where yellow and cyan represent Se and Mo atoms, respectively. (c) Photoluminescence spectrum.

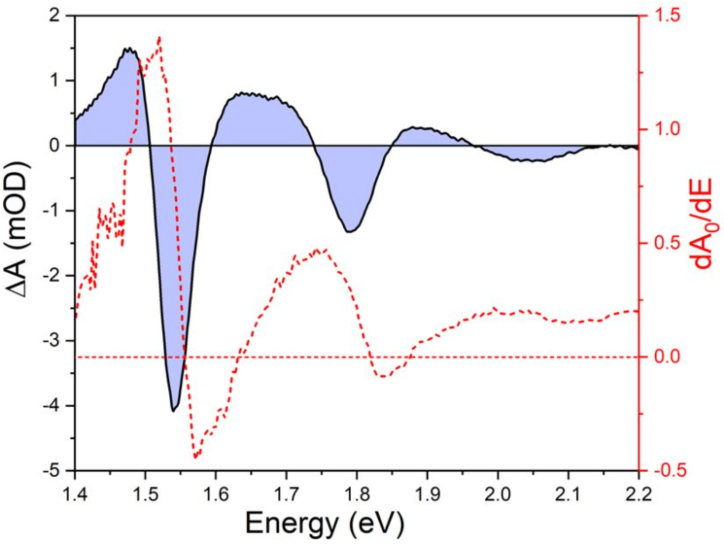

Figure S2. Comparison of TA spectrum (black-solid curve) at a time delay of 5 ps plotted against the left axis, with the first-order derivative of the ground-state absorption spectrum with respect to photon energy (red-dotted curve) plotted against the right axis.

\

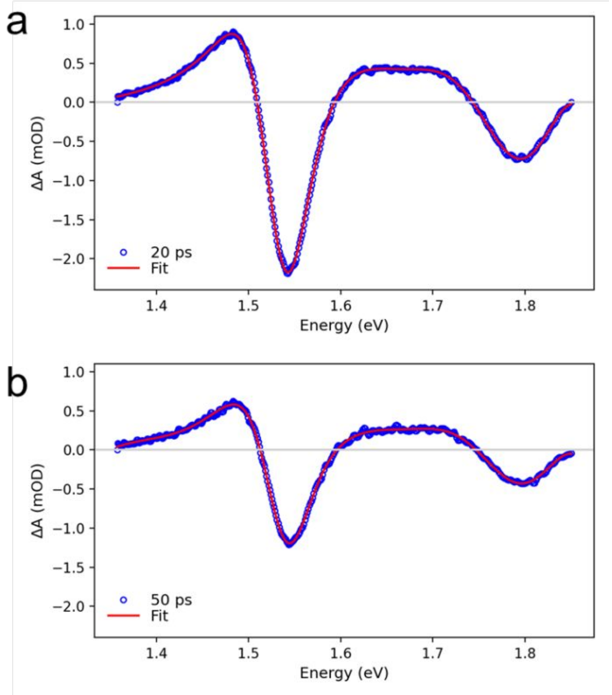

Figure S3. Examples showing the fit (red) of the TA spectra using the expression of $\Delta A(E,t)$ in terms of Lorentzian functions and fifth-order polynomials for the excited and non-excited sample, as described in the main text, obtained with a pump photon energy of 1.59 eV after (a) τ = 20 ps and (b) τ = 50 ps.

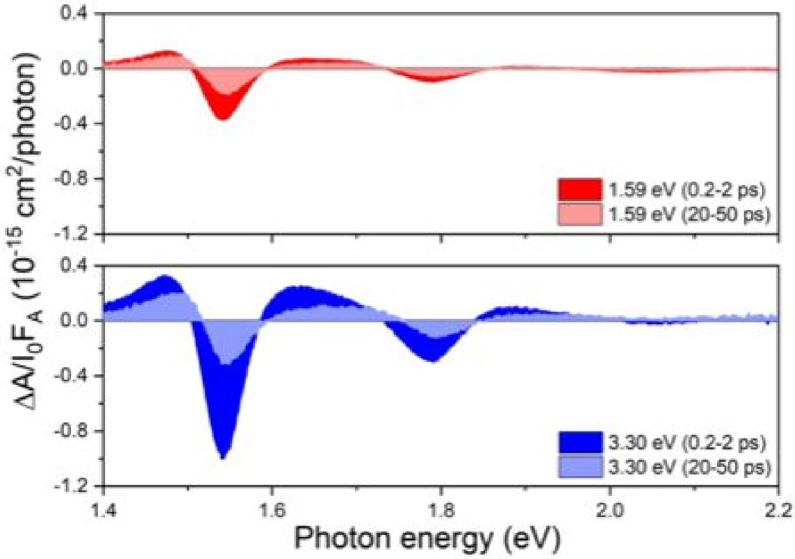

Figure S4. Transient change in optical absorption normalized to the absorbed fluence of pump photons, $\Delta A/I_0F_A$, after photoexcitation at 1.59 eV (upper panel) and 3.30 eV (lower panel) averaged over time intervals, as indicated. The fluences are the same as in Figure 3a. On short time the bleach near 1.54 eV is more pronounced than on longer time.

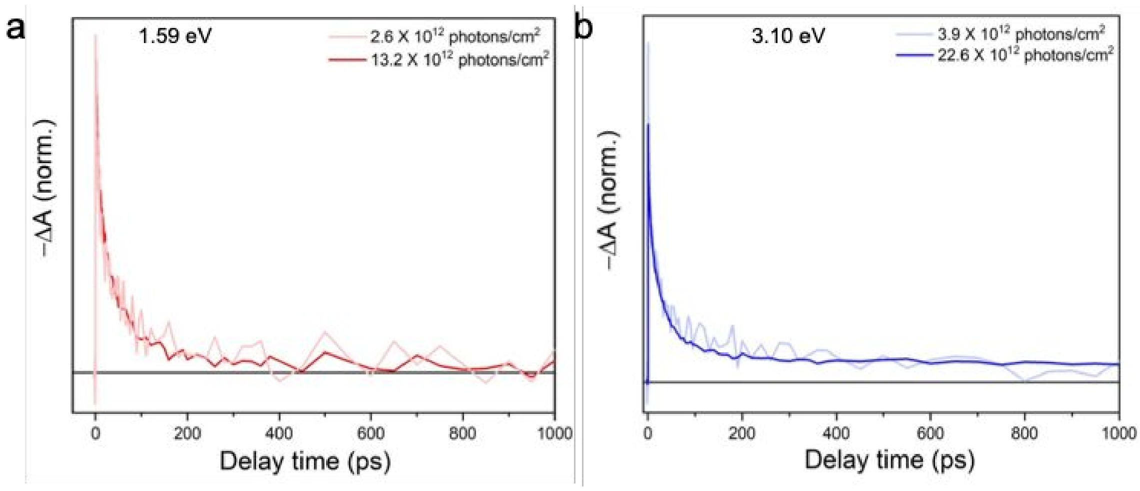

Figure S5. Decay kinetics of normalized TA signals probed at 1.54 eV for low and high absorbed pump fluences, following photoexcitation at (a) 1.59 eV and (b) 3.10 eV. The similar kinetics for low and high pump fluence implies that charge carriers decay by first-order electron-hole recombination or trapping rather than higher-order (Auger) processes.

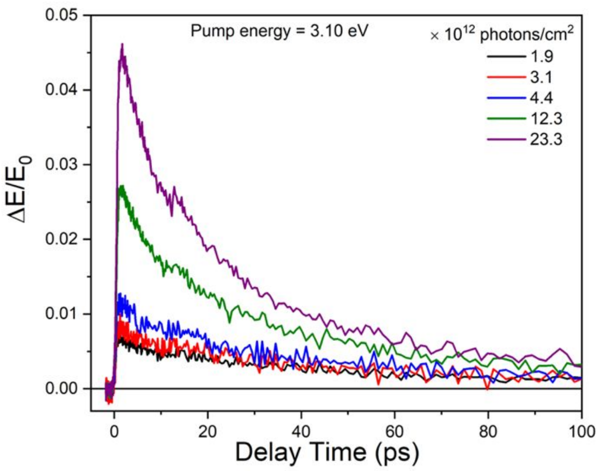

Figure S6. Pump fluence-dependent THz photoconductivity dynamics upon excitation at 3.10 eV.

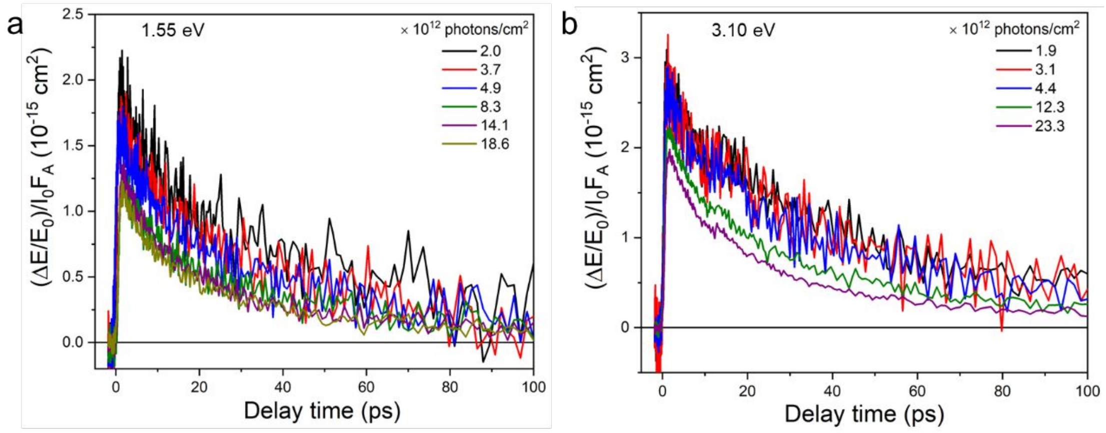

Figure S7. The THz photoconductivity dynamic normalized to the absorbed photon density, following photoexcitation at (a) 1.55 eV and (b) 3.10 eV.

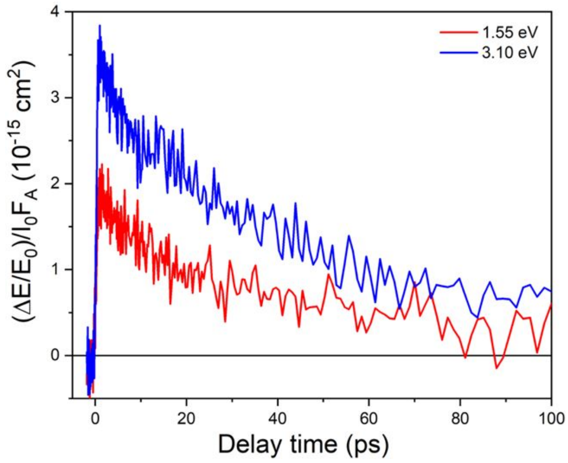

Figure S8. Comparison of photoconductivity dynamics following photoexcitation at 1.55 and 3.10 eV, normalized to an absorbed photon fluence of $2 \pm 0.1{\times}10^{12}$ photons/cm²

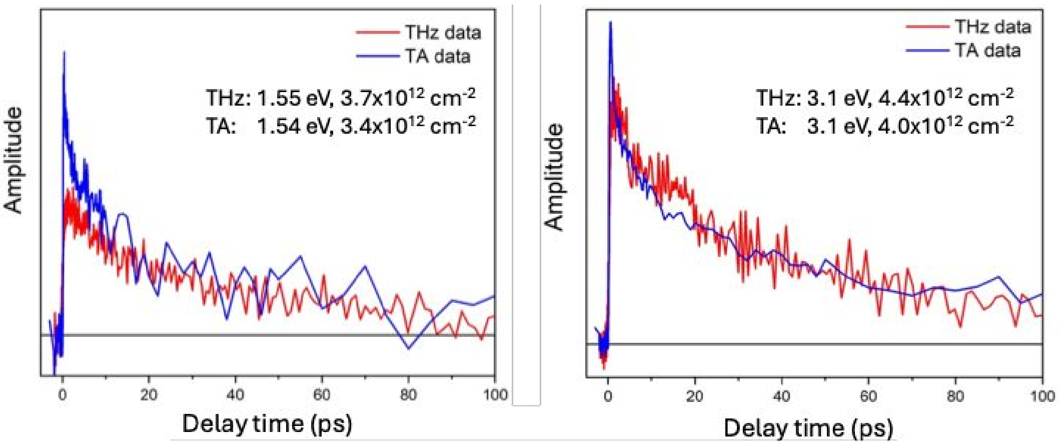

Figure S9. Comparison of decay kinetics THz (OPTP) and TA bleach (at 1.54 eV) signals after photoexcitation with low (left) and high (right) pump photon energies as indicated together with the absorbed pump fluence. The decays are similar after 20 ps. On short times after pump at low energy (left) the TA bleach is higher than the THz signal. This may be due to the initial formation of A excitons that lead to a bleach by probe induced photon emission. Decay of initially produced excitons and relaxation of primary energetic charge carriers to the band edges, or trapping, causes the THz and TA decay kinetics to eventually become similar.

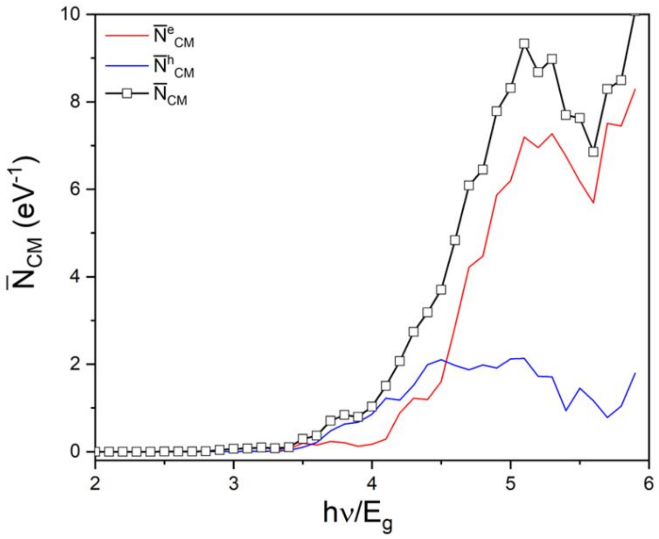

Figure S10. Calculated total summed average density of CM pathways, $\overline{N}_{CM}$, along with individual contributions from electrons ($\overline{N}^{e}_{CM}$) and holes ($\overline{N}^{h}_{CM}$), as a function of band gap multiple ($h\nu/E_g$). Here, $\overline{N}_{CM} = \overline{N}^{e}_{CM} + \overline{N}^{h}_{CM} = \frac{1}{N_{h\omega}}\sum_{i=1}^{N_{h\omega}}[N^{e_i}_{CM} + \ N^{h_i}_{CM}]$.

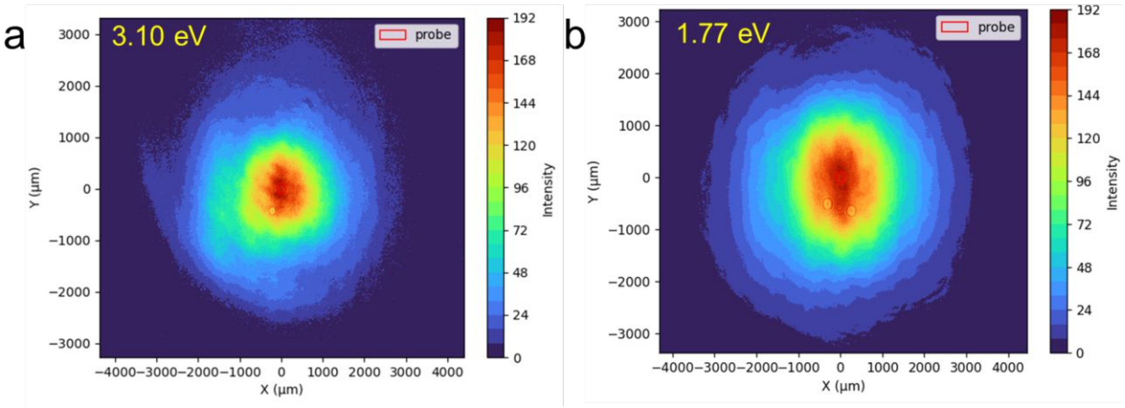

Figure S11. Representative beam profile images at pump photon energy of (a) 3.10 eV and (b) 1.77 eV.
